# Trachoma Rapid Assessments in Unity and Northern Bahr-el-Ghazal States, Southern Sudan

**DOI:** 10.1371/journal.pone.0013138

**Published:** 2010-10-01

**Authors:** Emily Robinson, Lucia W. Kur, Aggrey Ndyaba, Mounir Lado, Juma Shafi, Emmanuel Kabare, R. Scott McClelland, Jan H. Kolaczinski

**Affiliations:** 1 Malaria Consortium – Africa Regional Office, Kampala, Uganda; 2 Ministry of Health, Government of Southern Sudan, Juba, Southern Sudan; 3 Institute of Tropical and Infectious Diseases, University of Nairobi, Nairobi, Kenya; 4 Division of Allergy and Infectious Diseases, University of Washington, Seattle, Washington, United States of America; 5 Department of Disease Control, London School of Hygiene and Tropical Medicine, London, United Kingdom; Veterinary Laboratories Agency, United Kingdom

## Abstract

**Background:**

Trachoma is thought to be endemic over large parts of Southern Sudan, but empirical evidence is limited. While some areas east of the Nile have been identified as highly endemic, few trachoma surveys have been conducted in the remainder of the country. This study aimed to determine whether trachoma constitutes a problem to public health in Northern Bahr-el-Ghazal and Unity State, both located west of the Nile.

**Methods and Principal Findings:**

Trachoma rapid assessments (TRA) were conducted between July and September 2009. Seven villages in Northern Bahr-el-Ghazal State and 13 villages in Unity State were surveyed; an average of 50 children (age 1–9 years) and 44 women (age 15 years and above) were examined per village. Samples for analysis using the APTIMA Combo-2 nucleic acid amplification test (NAAT) were collected from participants with active trachoma in eight villages in Unity State. In Northern Bahr-el-Ghazal State, only three children with active trachoma (trachomatous inflammation follicular (TF) and/or trachomatous inflammation intense (TI)) and two women with trichiasis (TT) were found, in two of the seven villages surveyed. In Unity State, trachoma was endemic in all thirteen villages surveyed; the proportion of children with active trachoma ranged from 33% to 75% between villages, while TF in children ranged from 16% to 44%. Between 4% to 51% of examined women showed signs of TT. Samples from active trachoma cases tested using the NAAT were positive for *Chlamydia trachomatis* infection for 46.6% of children and 19.0% of women.

**Conclusions:**

Trachoma presents a major problem to public health Unity State, while the disease is of low priority in Northern-Bahr-el-Ghazal State. Implementation of a population-based prevalence survey is now required in Unity State to generate baseline prevalence data so that trachoma interventions can be initiated and monitored over time.

## Introduction

Trachoma, caused by ocular infection with the bacterium *Chlamydia trachomatis*, is the leading cause of infectious, preventable blindness worldwide, responsible for an estimated 3.6% of all cases of blindness [1]. The disease is endemic in 57 countries in Africa, the Middle East, Central and South America, Asia, Australia and the Pacific Islands. Globally an estimated 40.6 million people are living with active trachoma (trachoma inflammation-follicular (TF) and/or trachoma inflammation-intense (TI)) and 8.2 million with trachomatous trichiasis (TT) [2]. In 1997, the World Health Organization (WHO) established the Alliance for the Global Elimination of Blinding Trachoma by the year 2020 (GET 2020) to support country implementation of the SAFE strategy. SAFE stands for **S**urgical correction of trichiasis, **A**ntibiotics for treatment of active trachoma infection, **F**acial cleanliness and **E**nvironmental improvement [3], [4]. One of the challenges faced by trachoma control programmes is to cost-effectively target SAFE to communities most in need [5].

Trachoma endemic communities are generally located in remote rural areas of developing countries where access to water, sanitation and health care is inadequate [5], [6].

Geographical remoteness and inaccessibility mean that it is often difficult and costly to conduct fieldwork in these underserved areas; consequently there are limited data on the distribution of trachoma in many endemic countries [2], [7]. Southern Sudan provides one such example; large parts are thought to be trachoma endemic but data on its distribution and burden are mainly limited to areas east of the river Nile [8], [9]. Most of the surveys conducted to date reported prevalences of TF in children age 1–9 years well above 10% [8], thus exceeding the WHO recommended threshold for mass drug administration (MDA) of antibiotics [3]. However, areas with little to no trachoma have also been identified, demonstrating that by no means is all of Southern Sudan trachoma endemic [10]. Considerable heterogeneity in trachoma transmission is also indicated on a recently developed trachoma risk map of Southern Sudan [11]. There is thus a clear need for further trachoma surveys to confirm which parts of the country need to be targeted with SAFE interventions. Areas will be prioritized for intervention if the prevalence of active trachoma in children age 1–9 years is 10% or higher, or if the prevalence of trichiasis in people age 15 years and over is 1% or higher [3].

The trachoma rapid assessment (TRA) method, which uses a two-stage sampling design to optimally bias sampling toward communities most likely to have trachoma [12], provides a useful first step in categorizing areas for intervention [13]–[17]. TRAs have been successfully used to determine the presence of trachoma in a number of countries such as Ethiopia [18], Yemen [19], the Pacific islands [20] and India [21]. We decided to conduct TRAs in two states of Southern Sudan for which there were little (Unity State [22]) to no data (Northern Bahr-el-Ghazal State) on trachoma, but which are indicated to be at risk of trachoma transmission [11]. These are also two of the first states targeted by Southern Sudan's integrated programme for the control of key neglected tropical diseases [9], which provides opportunities to scale up trachoma control.

The purpose of the TRAs was to confirm whether trachoma is a public health problem in these two suspected endemic areas. In addition, we aimed to determine the viability of using the APTIMA Combo-2 nucleic acid amplification tests (NAAT) in a context such as Southern Sudan. The use of NAATs as an adjunct to clinical assessments has been suggested for monitoring the impact of antibiotic MDA, because antibiotic treatment has a more rapid impact on *C. trachomatis* infection levels than on symptoms of clinical disease [23]. The use of laboratory assays to accurately measure *C. trachomatis* prevalence before and after antibiotic MDA is therefore important for monitoring progress towards elimination once trachoma endemic areas have been identified.

## Methods

### Ethical considerations

The study protocol received ethical approval from the Directorate of Research, Planning and Health System Development, Ministry of Health (MoH), Government of Southern Sudan. Clearance to conduct the survey was also obtained from the respective State MoHs, followed by County Health Departments.

The study was explained to the village chief, household heads, and each adult participant. Household heads were asked to provide written consent for the household to participate in the study, by providing a thumbprint on the consent form after its contents were read out. If the household head was absent, the most senior person identified by household members was requested to provide written consent. Each adult participant provided verbal consent before participating in the study; for all children examined, consent was obtained from the guardian or household head. Households or individuals for whom informed consent was not given were not included in the survey. Household members that were unwell were not examined for trachoma, and examinations were halted if a person appeared to be in obvious distress. Personal identifiers were removed from the dataset before analysis.

### Study sites

**Figure 1:**
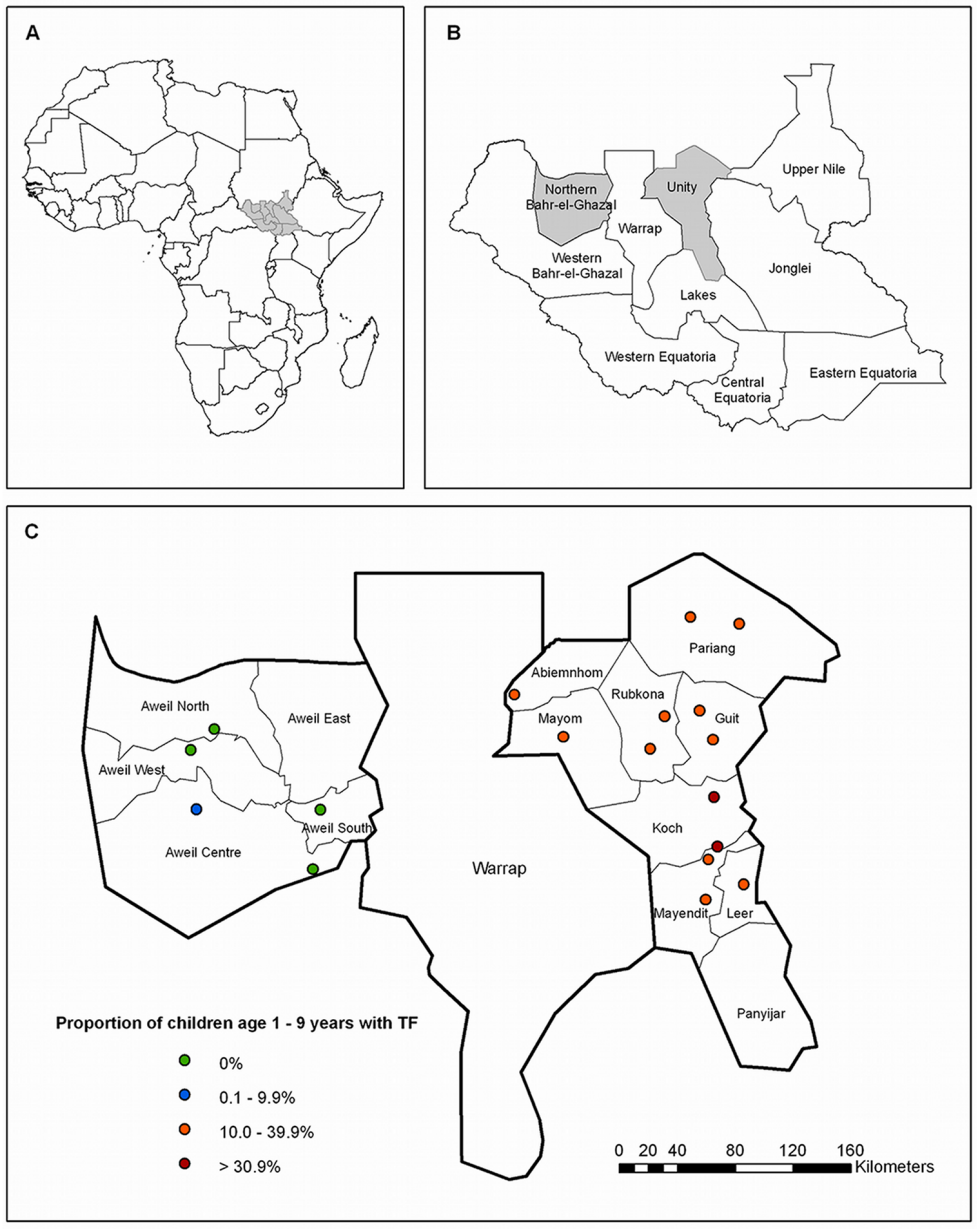
Location of survey area and survey sites. A) Map of Africa showing location of Southern Sudan (grey shaded); B) Map of Southern Sudan showing location of the two surveyed states, Northern Bahr-el-Ghazal and Unity (grey shaded); C) Detailed map of the surveyed states, including new county boundaries and the proportion of children age 1–9 years with TF in each surveyed village.

TRAs were conducted between July and September 2009 in Northern Bahr-el-Ghazal and Unity States, Southern Sudan (Figure 1 A & B). Northern Bahr-el-Ghazal State is divided into five counties with an estimated population of 720,898, the majority of whom are Dinka. Unity State is mainly inhabited by Nuer, and divided into nine counties with an estimated population of 585,801, according to census data collected in 2008. In Northern Bahr-el-Ghazal State, TRAs were conducted in seven villages distributed over five counties, while in Unity State a total of thirteen villages in eight counties were assessed (Note that revised county shape files recently released by the United Nations Office for the Coordination of Humanitarian Affairs in Juba, used to develop figure 1, are not entirely consistent with the location of villages as indicated by County Health Departments in 2009). In Unity State, Payinjar county could not be surveyed due to security and access issues; for the same reasons only one village was surveyed each in Mayom and Abiemnhom counties.

### Survey design

The TRA methodology used here has been described elsewhere [12]. The method was designed to quickly and cheaply generate “worst case estimates” on the endemicity of active trachoma and/or TT in a community and, by extrapolation, in a given administrative area (e.g. district); the method was not developed to provide estimates of prevalence. Based on TRA results it should be possible to exclude areas of no or low endemicity from further surveys and to target population-based prevalence surveys (PBPS) to suspected highly endemic areas, generating prevalence estimates to determine the actual need for control activities and allow their subsequent monitoring and evaluation.

To generate “worst case estimates” we used a convenience sampling methodology to identify counties and villages most likely to be endemic for trachoma. Initially, published and grey literature was reviewed, and anecdotal information was gathered through meetings with staff from the State MoH and County Health Department. The information thus obtained was used to categorize each county according to the likelihood of trachoma. In counties where there was no evidence of trachoma being endemic, one village was selected for the TRA, while two villages were selected in counties where there was evidence of transmission. Within each county, the villages most likely to have trachoma were prioritised for sampling. In counties with no information on trachoma, villages were chosen on the basis of known risk factors, such as poor access to water and sanitation [24]; in these counties sampling was stopped if no trachoma was found in the first village visited. Geographic areas recently surveyed for blinding trachoma [22] were excluded from the study.

The survey team was comprised of three permanent staff; a team leader, one ophthalmologist and one ophthalmic nurse. An additional two short-term staff to obtain consent from and register participants, and a driver, were hired in each state. Prior to the survey, sensitisation visits were conducted in each village. The study's purpose and methods were explained to the villagers, information on the transmission and cause of trachoma was provided, and photographs of trichiasis were shown. A questionnaire was used to conduct an interview with the village leader, to gather information on the presence of trachoma in the community and on various environmental and social risk factors relating to trachoma transmission. Permission to return and conduct the survey was requested from the village leader, who was also asked to mobilise the community and request women and children to gather at the village central-point at a specific time on the survey day.

On the survey day, participants were selected from those gathered at the central point. In most villages all women and children gathered were included in the study, in an attempt to enrol a total of 50 women age 15 years and above and 50 children age 1–9 years in each village. Before registration, each household head was asked how long their family had been living in the village; only those who had been resident for over six months were registered. Each study participant was assigned an identification number and her/his age and sex was recorded.

### Trachoma examination

Participants were examined for signs of trachoma either by an experienced ophthalmologist or an ophthalmic clinical officer, according to the WHO simplified grading system [25]. This scheme categorises trachoma infection according to five grades: TF, TI, trachomatous scarring (TS), TT and corneal opacity (CO). Before the start of the survey, the grading skills of the clinical officer were verified by the ophthalmologist using a two-stage assessment. In the first stage it was assessed whether the clinical officer was able to correctly identify at least 80% of slides in a given set [26]. Subsequently she was observed examining 50 women and 50 children in the field, to verify that at least 80% inter-observer agreement was reached with the ophthalmologist.

During the survey, participants had both eyes examined using a binocular loupe with 2.0× magnification and a torch. Each eye was initially examined for CO, as well as for in-turned lashes characteristic of TT. The upper tarsal conjunctiva was then examined for active trachoma (TF and/or TI) and TS. Trachoma signs only had to be present in one eye for the person to be categorised as suffering from a particular grade of trachoma. Alcohol-soaked cotton swabs were used to clean the examiner's fingers between individual examinations. The visual acuity of women age 15 years and above was measured using a tumbling E-chart.

Individuals with signs of active trachoma were treated with azithromycin tablets (for adults) or syrup (for those age five years or younger) and provided with information on face washing and good hygiene practices. Patients with TT or other significant eye conditions were referred to the nearest facility offering free eye surgery.

### Collection and analysis of NAAT samples

The APTIMA Combo 2 Unisex Swab Specimen Collection Kit (Gen-Probe, Inc., San Diego, CA, USA) was used for collection and analysis of samples to be tested by means of nucleic acid amplification. This type of NAAT detects rRNA, rather than DNA, which has been indicated to be more sensitive for detection of *C. trachomatis* infection [27], [28]. The overall sensitivity and specificity of the APTIMA Combo-2 assay for detection of *C. trachomatis* in genital tract specimens has been reported as 96.1% and 98.0% respectively [29]. The assay has also been validated for rectal, pharyngeal, and conjunctival samples, showing 100% agreement with confirmatory probe assays [30]. Prior studies in Africa have used this assay for determining the prevalence and treatment outcomes of trachoma [31].

Delivery of sample collection tubes to the field was delayed, which meant that samples could only be collected from the final eight villages surveyed in Unity State. In these villages, an eye swab was taken from the upper tarsal conjunctiva of one eye of each study participant with active trachoma, following the method described by Yang and colleagues [27]. Other than the presence of active trachoma in that eye, no specific method was used to decide whether the left or the right eye was examined; this decision was left to the ophthalmologist. Two types of negative field control were also collected in each village, from the first women and children examined with no signs of trachoma, in order to assess potential field contamination. Samples from two women and two children were taken for each control type. For ‘type-one’ controls, the swab was passed over the upper tarsal conjunctiva; for ‘type-two’ controls the swab was passed 2.5 cm above, but not touching, the conjunctiva. The samples were stored in collection tubes at room temperature and processed within 60 days of collection. Samples were processed according to the manufacturer's instructions. An internal quality-control process was conducted during which 126 positive samples and 16 negative samples were re-tested. The positive samples that were re-tested were trace positive samples that abutted fully positive samples. The negative samples that were re-tested were randomly selected from all the negative samples. The repeat-tests were conducted by the same individuals using the same type of assay.

### Data management and analysis

Data were verified and entered into the study database, created in Microsoft® Office Excel®2007 at the end of each day, and double entered after completion of the survey. Range and consistency checks were conducted for all variables. Data were analysed in STATA 9.0 (Stata Corporation, College Station, TX, U.S.A.). The proportion of those examined with each grade of trachoma and associated 95% confidence intervals were calculated for children and adults examined at each of the survey sites.

## Results

A total of 331 children and 278 women were examined in Northern Bahr-el-Ghazal State, and 671 children and 605 women in Unity State. The number of children and women examined in each village averaged 47 and 40 in Northern Bahr-el-Ghazal State and 51 and 47 in Unity State, respectively. The average number examined in Northern Bahr-el Ghazal State was lower, because the residents of one village had been poorly mobilized; only 14 women and 29 children could thus be examined. Information on active trachoma in children could not be obtained for three children (one in Northern Bahr-el-Ghazal and two in Unity State), who refused to have their second eye examined. Information regarding presence of TT was recorded for all women examined.

In Northern Bahr-el-Ghazal State, trachoma cases were identified only in two of the seven villages surveyed (Table 1). One village, in Aweil Centre county, had three cases of active trachoma in children (i.e. 6% of study participants (95%CI, 1.25%–16.55%), two of which had TF (Figure 1 C). In the same village a 55 year old woman showed signs of TT. A second TT case was identified in a village in Aweil West county, in a woman age 65 years.

**Table 1:** Prevalence trachoma signs in Northern Bahr-el-Ghazal State.

County	Payam	Village name	Children age 1–9 years					Women age 15 years and above		
			No. examined	% active trachoma*	95% CI	% TF	95% CI	No. examined	% TT	95% CI
Aweil Centre	Barmayen	Kabat	29	0	0.0–11.9	0	0.0–11.9	14	0	0.0–23.2
	Chol South	Chamang Moui	50	6	1.3–16.6	4	4.9–13.7	50	2	0.1–10.7
Aweil East	Madhol	Atuet	50	0	0.0–7.1	0	0.0–7.1	50	0	0.0–07.1
Aweil West	Mayom	Akuak Rel	50	0	0.0–7.1	0	0.0–7.1	32	0	0.0–10.9
	Ayat	Malek Dira	50	0	0.0–7.1	0	0.0–7.1	34	2.9	0.1–15.3
Aweil North	Malual North	Mayom Adhal	49	0	0 0–7.3	0	0.0–7.3	48	0	0.0–7.4
Aweil South	Wathmuok	Majok	52	0	0.0–6.8	0	0.0–6.9	50	0	0.0–07.1

*TF and/or TI.

In Unity State, 33% (95%CI, 20.40%–48.41%) to 75% (95%CI, 61.63%–85.61%) of children examined in each village showed signs of active trachoma (Table 2). The prevalence of TF ranged from 16% (95%CI, 7.02%–28.59%) to 44% (95%CI, 30.47%–58.67%) between survey villages (Figure 1 C). Of women examined, 4% (95%CI, 0.53%–14.84%) to 51% (95% CI, 37.07%–64.65%) showed signs of TT. Four cases of TT in children age 1–9 years were observed in Unity State; the youngest child was seven years old.

**Table 2:** Prevalence of trachoma signs in Unity State.

County	Payam	Village name	Children age 1–9 years					Women age 15 years and above		
			No. examined	% active trachoma*	95% CI	% TF	95% CI	No. examined	% TT	95% CI
Rubkuona	Biel	Chambarou	48	33.3	20.4–48.4	22.9	12.03–37.3	36	19.4	8.2–36.0
	Pakur	Pakur	51	56.9	42.3–70.7	15.7	7.02–28.6	51	19.6	9.8–33.1
Koch	Ngony	Riang1	52	65.4	50.9–78.0	44.2	30.47–58.7	44	38.6	24.4–54.5
	Jaak	Phangak	57	66.7	52.9–78.6	40.4	27.56–54.2	46	45.7	30.9–61.0
Guit	Kadet	Kadet	56	51.8	38.0–65.3	28.6	17.30–42.2	46	4.4	0.5–14.8
	Nyathor	Nyathor	54	57.4	43.2–70.8	29.6	17.98–43.6	43	18.6	8.4–33.4
Mayandit	Rupqui	Dorinyet	56	75.0	61.6–85.6	26.8	15.83–40.3	53	26.5	15.3–40.3
	Bhou	Bhou	48	47.9	33.3–62.8	25.0	13.64–39.6	46	6.5	1.7–17.9
Ruweng	Jam Jany	Mankuor	50	48.0	33.7–62.6	18.0	08.58–31.4	42	33.3	19.6–49.6
	Nyel	Kamagon	50	60.0	45.2–73.6	22.0	11.53–36.0	44	22.7	11.5–37.8
Abiemnhom	Manajoga	Wungok	49	42.9	28.8–57.8	28.6	16.58–43.3	50	24.0	13.1–38.2
Mayom	Wanguara	Pibor	48	56.3	41.2–70.5	33.3	20.40–48.4	49	20.4	10.3–34.3
Leer	Adok	Thor	50	48.0	33.7–62.6	16.0	7.17–29.1	55	50.9	37.1–64.7

*TF and/or TI.

No visual acuity data were obtained for one (0.4%) of the study participants in Northern Bahr-el-Ghazal State and for 70 (11.6%) of participants in Unity State. The majority (n = 57) of instances where visual acuity was not recorded occurred in two villages in Unity State (Bhou in Mayandit county and Pakur in Rubkuona county) and was due to time constraints and a limited team size. In two of the villages surveyed in Northern Bahr-el-Ghazal State, none of the women examined had bilateral visual impairment (VI) or blindness, while the proportion of women with bilateral VI in the remaining villages ranged from 4.0% (95%CI, 0.49%–13.71%) to 42.9% (95%CI, 28.8%–57.8%), and with bilateral blindness ranged from 4.0% (95%CI, 0.5%–13.7%) to 24.5% (95%CI, 13.3%–38.9%).

In Unity State, bilateral VI ranged from 15.6% (95%CI, 6.5%–29.5%) to 51.9% (95%CI, 37.8%–65.7%). In two of the villages sampled, none of the women had bilateral blindness. In the remaining eleven villages, 2.2% (95%CI, 0.1%–11.5%) to 18.5% (95%CI, 9.3%–31.4%) of women examined suffered from bilateral blindness.

### NAAT results

Eye swabs were taken from 95% (208/220) of children with active trachoma, and 95% (84/88) of women with active trachoma (TF and/or TI) in eight villages in Unity State. Of these, 46.6% (n = 97) of children and 19.0% (n = 16) of women were positive for *C. trachomatis* (Table 3). Control type-one samples were taken from 24 children and 18 women, randomly selected from individuals with no signs of active trachoma. Of the 24 control type-one samples from children and the 18 control type-one samples from women, 12.5% (n = 3) and 11.1% (n = 2) tested positive for *C. trachomatis*, respectively. 27 type-two control samples were also collected; 14 from women and 13 from children. None of these samples were positive for *C. trachomatis*.

**Table 3:** Results from clinical diagnosis of active trachoma compared to NAAT.

Clinical examination result	Children age 1–9 years			Women age ≥15 years			Total No.
	NAAT +ve No. (%)	NAAT −ve No. (%)	Total No.	NAAT +ve No. (%)	NAAT −ve No. (%)	Total No.	
Active trachoma* +ve	97 (46.6)	111 (53.4)	208	16 (19.0)	68 (81.0)	84	292
Active trachoma* −ve (Control type 1)	3 (12.5)	21 (87.5)	24	2 (11.1)	16 (88.9)	18	42
Control type 2	0 (0.0)	13 (100.0)	13	0 (0.0)	14 (100.0)	14	27

*TF and/or TI.

Of the 292 individuals with active trachoma tested using the APTIMA Combo-2 NAAT, 34.0% (n = 105) and 77.4% (n = 226) had been diagnosed with clinical signs of TF or TI, respectively. Of the individuals with TF, 36.9% (31/84) of the children and 4.8% (1/21) of the women were positive for *C. trachomatis* as identified by the NAAT. Of the individuals diagnosed with TI, 54.4% (81/149) of the children and 20.1% (16/77) of the women tested positive for *C. trachomatis* with the NAAT. Re-testing confirmed the accuracy of the results, as no false positive or false negatives were found.

## Discussion

The present study set out to determine whether trachoma is a public health problem in two states of Southern Sudan – Northern Bahr-el-Ghazal and Unity – and hence provide guidance to the national trachoma programme on where to target its scarce resources. If the TRA results are taken as estimates of prevalence they indicate that all communities surveyed in Unity State exceeded TF prevalence in children age 1–9 years of 10%, at which level WHO recommends MDA of antibiotics [3]. This finding is consistent with results from a PBPS in Mankien payam, Mayom county, which reported 57.5% prevalence of TF in children [22] and with the predictions of a trachoma risk map of Southern Sudan [11]. It is not possible to draw conclusions regarding trachoma prevalence at state or county level based on the present results, because survey sites and individuals within each site were not selected at random and because too few sites were surveyed in each administrative unit [32], [33]. A PBPS is therefore now required in Unity State to generate baseline data on trachoma prevalence prior to scaling up of SAFE interventions, so that the trachoma control programme can be monitored and evaluated over time.

Northern Bahr-el-Ghazal State on the contrary is not a priority area for the trachoma control programme, as the low levels of trachoma found by the TRA indicate that the disease is not a major public health problem in this state. All villages visited had been identified by local health authorities as the ones most likely to have trachoma and it is therefore considered unlikely that any foci of active trachoma and/or TT were missed. The TRA results contrast with climate-based predictions of the above mentioned risk map [11], which indicated that at least some parts of Northern Bahr-el-Ghazal State may be at risk of trachoma transmission. This finding highlights an important point made elsewhere, but often forgotten; the primary use of risk maps is to identify likely areas of no or low transmission and then exclude these from further surveys or intervention unless evidence to the contrary emerges [34]. Risk maps on their own are not to be used to target trachoma interventions. To be useful tools, trachoma risk maps need to be validated locally, and should only be used in conjunction with other sources of evidence prior to excluding communities from interventions. Despite the existence of a trachoma risk map for Southern Sudan, verification of suspected at-risk areas through TRAs is therefore an essential step in the process of targeting trachoma control. If TRA results indicate that trachoma is likely to be meso- to hyper-endemic (as in the case of Unity State), PBPS should then be conducted to generate baseline data prior to SAFE intervention. In the case of Southern Sudan, the prevalence data generated by the PBPS can then be incorporated into the risk mapping model, further improving its predictions [11].

While no household-level information regarding trachoma risk factors was collected, various possible explanations for the observed difference in trachoma endemicity between Unity and Northern Bahr-el-Ghazal State may be considered. Cattle ownership, face-washing practices and high household fly densities have been identified as trachoma risk factors [24], [35], and these clearly differed at community level between the two states. Village elders interviewed during community sensitization reported pastoralism as the main occupation in Unity State, but only in three of the seven villages surveyed in Northern Bahr-el-Ghazal State. This difference in cattle ownership is likely to be a reflection of the different tribal compositions in each state: Northern Bahr-el-Ghazal State is primarily inhabited by Dinka who generally rely on both cattle and agriculture as sources of income, whereas Unity is largely inhabited by the Nuer tribe, which relies more heavily on a pastoralist livelihood. This reported difference in livelihoods was confirmed through field observations; compared to villages in Northern Bahr-el-Ghazal State family compounds in Unity State contained noticeably more cattle, as well as flies, and families were often found to be drying cattle dung inside their compounds, reportedly to burn it as a mosquito repellent. Access to clean water was also reported to differ, with all but one of the villages in Northern Bahr-el-Ghazal State having access to a hand pump, compared to only four of the 13 villages visited in Unity State. Although these results do not allow any definitive conclusions on the correlation of potential risk factors and trachoma prevalence, they are consistent with results from an in-depth study that included data from Unity State [24].

While prevalence data on clinical signs of trachoma will be required before SAFE intervention is scaled up in Unity State and elsewhere, combination of clinical assessments with laboratory results confirming the presence or absence of *C. trachomatis* will become of increasing importance for monitoring of programme impact. Once antibiotic MDA is being scaled up, it is expected to rapidly decrease infection levels while the decline in symptoms of clinical disease will be more gradually. Clinical assessments on their own would thus underestimate the actual impact on trachoma transmission.

In the present study, *C. trachomatis* was confirmed in 38.4% of all active trachoma cases sampled. This relatively low correlation between clinical diagnosis and NAAT result is consistent with other studies and likely to be due to the kinetics of the disease, rather than problems with the NAAT detection system [5], [23]. Trachoma has a four to seven day latent phase during which patients are infected but are not showing clinical signs. Infection then cause patent disease of variable duration, with both infection and clinical signs being present. After infection has been cleared, clinical signs slowly resolve over a recovery phase that can take several years [36]. Our result may therefore be at least partially explained by the fact that some of the examined patients will have been in the recovery phase, showing clinical signs of trachoma while being no longer infected with *C. trachomatis*. In addition, in some the patients the observed follicles may not have been a sign of *C. trachomatis* infection, but may have been caused by other bacteria, such as *Moraxella spp*. and *Streptococcus pneumonia*, or by viral infections, such as adenovirus, *Molluscum contagiosum* or herpes simplex [37], [38].

As demonstrated by the presence of positive NAAT results in the type-one control samples it is also possible for *C. trachomatis* to be detected in the eyes of individuals that do not show signs of active trachoma. It is unlikely that this observation is the results of laboratory contamination, as our internal quality control process identified no false-positives. It is also unlikely to be the result of contamination of the samples in the field, because none of the control type-two samples were positive. Probable explanations are that individuals from which samples were tested with the APTIMA Combo-2 NAAT were in the latent phase of the disease or had low-level, asymptomatic, infections. The NAAT result may also have been positive because infection detectable with the RNA-based assay used here can persist in the tarsal conjunctiva after clinical disease has resolved [23], [39], [40]. Lastly, the criteria for clinical diagnosis under the simplified grading system are fairly narrow; patients with follicles present outside the central part of the upper tarsal conjunctiva or with fewer than five follicles are not considered as having signs of trachoma, while they may be infected with the bacteria [25], [37].

The present study had several limitations that may have led to over or under-estimation of the trachoma problem. Overestimation may have occurred as a result of purposefully selecting villages to be likely to have trachoma, as recommended by the TRA methodology [12]. The proportions of individuals with trachoma signs in the present study villages are therefore likely to be higher than the overall prevalences for each county. Sensitization visits to survey villages before the survey and the use of central point registration may also have resulted in an overestimate of trachoma signs, particularly TT and CO, as women with these eye problems may have been keen to get treatment and hence been more likely to participate in the survey than those with no eye conditions [17]. Given that TRAs were designed to provide a worst-case estimate of the trachoma problem in a particular area, these implications of the study design are generally well understood [17]. However, TRA methodology also relies anecdotal information on the presence of trachoma to assist with the identification of villages to be assessed. Unfortunately we found that the understanding of trachoma signs and symptoms was often limited, even among health workers, which may have led to exclusion of highly trachoma endemic villages from the TRA, in turn leading to an underestimate of the problem. Such underestimate may have also resulted from lack of accessibility; fieldwork was conducted during the rainy season, meaning that many villages could not be reached. It is possible that these inaccessible villages had worse access to healthcare and clean water and may therefore have been more affected by trachoma.

### Conclusion

Unity State should be prioritized for further trachoma interventions. Initially a PBPS will be required to generate baseline prevalence data, to allow monitoring and evaluation of subsequent intervention. The survey should be accompanied by administration of household questionnaires, to get a better understanding of the environmental and behavioural risk factors for trachoma in this primarily Nuer-inhabited area. In Northern Bahr-el-Ghazal State trachoma is not a major public health problem, but efforts should be made to provide antibiotic treatment to individuals with signs of active trachoma on a case-by-case basis and to provide access to surgery for patients with trichiasis. The northern part of Warrap State, which lies between Unity and Northern Bahr-el-Ghazal States, would benefit from a TRA to provide insight as to how far trachoma extends to the west from Unity State.

Collection of samples to be examined with the APTIMA Combo-2 NAAT is feasible in the context of Southern Sudan, largely because no cold chain is required if samples are processed within 60 days. However, within this period samples need to be shipped to an adequately equipped laboratory, which is currently not available in Southern Sudan. Conducting NAATs such as the APTIMA does, however, considerably increase survey expenses. With the kit used here, the costs of a swab collection tube and processing of each sample was about US$ 1.25 and US$ 20, respectively. These costs could, however, be reduced if samples were pooled for analysis [41]. While a NAAT is likely to be a useful complementary tool, both for evaluation of the impact of antibiotic MDA and to assess the requirement of MDA in hypo-endemic areas, the use of such type of test should be carefully assessed in the context of present resource constraints – for now resources may be better used by conducting further TRAs aimed at establishing the scale of the trachoma problem in Southern Sudan.
